# First-in-human study on the pharmacokinetics, safety, and tolerability of single escalating doses and multiple doses of XT1061, a novel core protein allosteric modulator, in healthy Chinese subjects

**DOI:** 10.3389/fphar.2026.1860210

**Published:** 2026-06-30

**Authors:** Qingmei Li, Yue Hu, Dandan Wu, Wenbo Zhen, Hong Zhang, Qi Xu, Haifeng Zhang, Jiangkai Sun, Mengchi Li, Yanhua Ding, Qinglong Jin

**Affiliations:** 1 Department of Pediatric Nephrology, Children’s Medical Center, The First Hospital of Jilin University, Jilin, China; 2 Phase I Clinical Research Center, The First Hospital of Jilin University, Jilin, China; 3 Xi’an Xintong Pharmaceutical Research Co., Ltd., Xi’an, China; 4 School of Nursing, Xi’an Jiaotong University Health Science Center, Xi’an, China; 5 Department of Hepatology, The First Hospital of Jilin University, Jilin, China

**Keywords:** clinical trial, core protein allosteric modulator, HBV, pharmacokinetics, XT1061

## Abstract

**Objective:**

XT1061 is a newly developed oral small-molecule agent designed to promote the assembly of empty capsids devoid of pregenomic RNA, representing a potential therapeutic approach for chronic hepatitis B (CHB). This first-in-human trial aimed to assess the pharmacokinetic profile and tolerability of single ascending doses and multiple doses of XT1061 in healthy Chinese individuals.

**Methods:**

The Phase Ia clinical study consisted of two components: a double-blind, randomized, placebo-controlled, single-ascending-dose assessment conducted under fasting conditions across dose levels ranging from 12.5 mg to 600 mg—including a food-effect evaluation at 62.5 mg—followed by a multiple-dose regimen at 250 mg administered under fasting conditions.

**Results:**

XT1061 demonstrated good tolerability in healthy Chinese participants, with no notable difference in the incidence of adverse events between the XT1061 and placebo groups. Following administration, the median time to peak concentration ranged from 1.0 to 2.5 h, while the mean elimination half-life varied between 2.864 and 11.478 h. Systemic exposure exhibited an approximately dose-proportional increase. Steady-state concentrations were achieved within approximately 2 days of repeated dosing, with mean accumulation indices ranging from 1.043 to 1.333. Additionally, concomitant food intake reduced the peak plasma concentration by approximately 50%, although it had no significant impact on total systemic exposure, as measured by the area under the curve.

**Conclusion:**

These findings indicate that XT1061 possesses a favorable safety profile and predictable pharmacokinetics, providing a solid foundation for advancing into further clinical investigations to evaluate its efficacy and safety in patients with CHB.

**Clinical Trial Registration:**

[http://www.chinadrugtrials.org.cn/index.html], identifier [CTR20232071].

## 1 Introduction

Chronic hepatitis B virus (HBV) infection affects approximately 296 million individuals globally and remains the primary cause of cirrhosis and hepatocellular carcinoma worldwide. The burden of chronic hepatitis B (CHB) is particularly high in regions across Asia and sub-Saharan Africa (Nartey et al., 2022; Azzam et al., 2023; Dusheiko et al., 2023; Hsu et al., 2023; Liu et al., 2023; Chen et al., 2024; Quintas et al., 2024). Evidence from prior research indicates that CHB can progress to liver cirrhosis and hepatocellular carcinoma, significantly increasing the risk of liver-related mortality (Chen et al., 2024).

Despite the availability of nucleoside or nucleotide analogs and pegylated interferon, which can effectively suppress HBV replication, current treatment options for HBV infection remain suboptimal (Chien and Liaw, 2022). Only a small fraction—fewer than 10%—of patients with CHB achieve functional cure, defined as hepatitis B surface antigen (HBsAg) loss and/or seroconversion, which is considered the optimal therapeutic goal (Chien and Liaw, 2022; Yao et al., 2025). Consequently, there is an urgent need to investigate novel therapeutic approaches. Developing new antiviral agents with improved efficacy and reduced toxicity is crucial to advancing the treatment outcomes for individuals with CHB(Dusheiko et al., 2023).

HBV core protein plays a pivotal role in the packaging of the viral genome. Small-molecule antiviral agents that target the HBV core protein interfere with viral replication by blocking the encapsidation of pregenomic RNA during capsid formation (Zhou et al., 2017; Corcuera et al., 2018; Yao et al., 2025). This disruption results in the production of nonfunctional or empty capsids, impairing the viral life cycle and contributing to a reduction in covalently closed circular DNA (cccDNA) levels during infection (Mak et al., 2017; Zhou et al., 2017; Corcuera et al., 2018; Fanning et al., 2019; Rat et al., 2019; Yuen et al., 2020; Zoulim et al., 2020; Zhang et al., 2021; Zoulim et al., 2022; Jia et al., 2023; Jiang et al., 2023). Therefore, core protein allosteric modulators are likely to be an important component of future combination therapy for chronic hepatitis B. XT1061, a novel oral small-molecule agent independently developed by Xi’an Xintong Pharmaceutical Research Co., Ltd., induces the formation of empty capsids devoid of pregenomic RNA, thereby inhibiting HBV replication. Currently, several oral small-molecule agents that induce the production of nonfunctional or empty capsids have been developed and studied in early-phase clinical trials, such as GLS4, ABI-H0731, JNJ-6379, GST-HG141, and RO7049389. All of these agents are in Phase I–III clinical trials, and there are currently no approved anti-hepatitis B drugs targeting this specific mechanism. The above-mentioned similar drugs demonstrated an average reduction of approximately 2–3 log_10_ IU/mL in HBV DNA compared to baseline at the end of 4 weeks of treatment in patients with chronic hepatitis B, confirming the therapeutic potential of this target against HBV (Yuen et al., 2020; Zoulim et al., 2020; Yuen et al., 2021; Zhang et al., 2021; Wu et al., 2024).

After 28 days of continuous XT1061 administration in mice, serum HBV DNA levels were significantly reduced. When combined with nucleoside analogs, XT1061 demonstrated synergistic antiviral activity. The human equivalent dose (HED) was estimated using the body surface area method. In dogs, the no-observed-adverse-effect level (NOAEL) was established after 28 days of continuous XT1061 administration, and the corresponding HED—applying a 10-fold safety factor—was calculated to be 87.31 mg. The HED derived from rat studies was 4,800 mg (also applying a 10-fold safety factor) (Zhang et al., 2022). In a mouse efficacy model, the effective dose of XT1061 monotherapy was 50 mg/kg, which translated to an estimated human effective dose of approximately 267 mg (unpublished data).

As this Phase Ia clinical trial of XT1061 represents the initial study conducted in China to assess its safety profile, tolerability, and pharmacokinetic characteristics in healthy Chinese individuals, the results will provide critical support for advancing to a Phase Ib clinical trial aimed at further evaluating the therapeutic efficacy of XT1061 in patients with CHB.

## 2 Materials and methods

### 2.1 Participants

A total of 90 subjects were enrolled in this study, among whom 1 subject voluntarily withdrew before drug administration. The remaining 89 subjects completed the study between 19 July 2023 and 30 April 2024. This first-in-human trial was designed to assess the pharmacokinetics, safety, and tolerability of single ascending doses and multiple doses of XT1061 under fasting and fed conditions.

The main inclusion criteria were: subjects (including their partners) were willing to avoid pregnancy from screening through 6 months after the last administration of the study drug and agreed to use effective contraception; aged 18–65 years; body mass index between 18 and 28 kg/m^2^; and normal physical examination findings and vital signs—or abnormal findings deemed clinically insignificant. The main exclusion criteria were: use of any prescription drugs, over-the-counter medications, vitamins, herbal products, or alcohol within 14 days before study drug administration; consumption of foods known to affect drug metabolism (e.g., grapefruit or mango), vigorous exercise, or other factors potentially influencing drug absorption, distribution, metabolism, or excretion within 14 days before study drug administration; or a positive screening test for hepatitis B surface antigen, hepatitis C antibody or core antigen, HIV antigen/antibody, or *Treponema pallidum* antibody.

### 2.2 Study design

This was a randomized, double-blind, placebo-controlled, ascending-dose study (Chinese Drug Trial Identifier: CTR20232071). The study employed a sentinel enrollment approach: two subjects—one male and one female—were enrolled initially and received the investigational product. After 72 h of observation (i.e., post-tolerance evaluation on Day 4), if no intolerable adverse events occurred, the remaining 8 subjects were enrolled and randomly assigned to receive the investigational product or placebo in a 3:1 ratio for single-ascending-dose (SAD) administration; alternatively, the remaining 9 subjects were enrolled and randomly assigned in a 2:1 ratio for multiple-dose administration.

**FIGURE 1:**
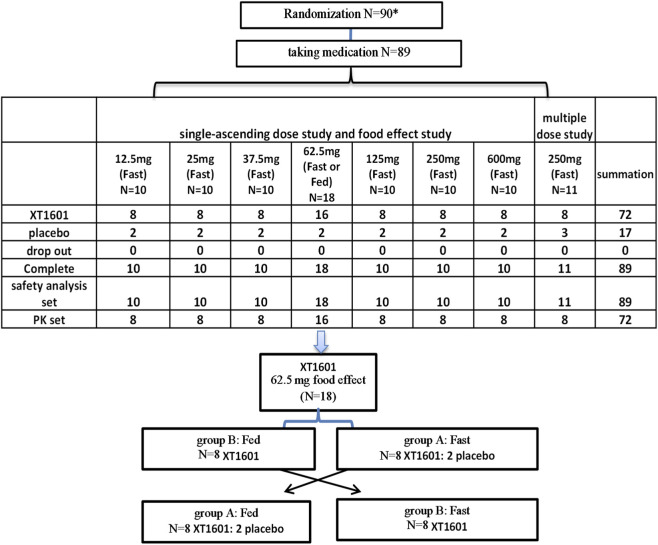
Flowchart depicting the variables examined in the study. * Subject No. 0908 voluntarily withdrew before drug administration.

This study fully complies with the CONSORT guidelines. The study protocol received ethical approval from the Ethics Committee of The First Hospital of Jilin University (Changchun, Jilin, China). The research was carried out in accordance with the principles outlined in the Declaration of Helsinki and adhered to the International Conference on Harmonization’s Good Clinical Practice standards, while also meeting applicable local regulatory criteria. Written informed consent was obtained from all participants before they were enrolled in the study. The study’s procedural timeline is illustrated in Figure 1.

#### 2.2.1 SAD group and food-effect assessment

A total of 70 healthy volunteers were randomly allocated into seven SAD groups (12.5 mg, 25 mg, 37.5 mg, 62.5 mg, 125 mg, 250 mg, and 600 mg), with each group comprising 10 participants (XT1061 to placebo ratio of 8:2). All groups received the study drug under fasting conditions, except for the 62.5 mg group. Safety assessments were performed on Day 2 and Day 4 following administration. Dose escalation proceeded sequentially, with enrollment into the next dose level contingent upon confirmation of acceptable tolerability in the preceding group.

To evaluate the impact of food intake on the pharmacokinetics (PK) of XT1061, a separate food-effect assessment was conducted within the 62.5 mg group involving 18 subjects (XT1061: placebo = 16:2). This assessment followed a randomized, two-period, two-sequence crossover design, in which participants received a single dose under both fasting and fed conditions. The metabolism of XT1061 was investigated under fasting conditions using fecal and urinary samples. In the fed arm, subjects ingested a high-fat, high-calorie meal (containing approximately 50% fat and providing 800–1,000 kcal) approximately 30 min prior to dosing. A 7-day washout period was implemented between treatment periods. Tolerability evaluations were conducted post-dose on Days 2, 4, 9, and 11.

#### 2.2.2 Multiple-dose group

A total of 11 healthy volunteers (randomized in a ratio of 8:3 to receive XT1061 or placebo) were initially enrolled in the multiple-dose phase to assess the safety and tolerability of XT1061 administered at a dose of 250 mg once daily for seven consecutive days. In this phase, tolerability was monitored following drug administration on Days 2, 4, 7, and 10, with all subjects maintained under fasting conditions throughout the study period.

The investigational product was administered orally with 240 mL of water. Both XT1061 and the matching placebo were developed and supplied by Xi’an Xintong Research Co., Ltd. The formulations were provided in capsule form, available in strengths of 12.5 mg and 50 mg, with corresponding batch numbers #230603/230601 and #230604/230602, respectively.

### 2.3 PK analysis

Venous blood (3 mL) was collected and transferred into tubes containing K_2_EDTA as an anticoagulant. After centrifugation (1,500 × g, 2 °C–8 °C, 10 min), plasma was separated and stored at −80 °C until analysis. Blood collection time points for the SAD group: 0 h (within 30 min before dosing), and 15, 30, 45 min, 1, 1.5, 2, 3, 4, 5, 6, 8, 12, 24, 48, and 72 h post-dose. Blood collection time points for the food-effect group: identical to those of the SAD group on Day 1 and Day 8.

Blood collection time points for the multiple-dose group: 0 h (within 30 min before the first dose on Day 1), and 15, 30, 45 min, 1, 1.5, 2, 3, 4, 5, 6, 8, and 12 h post-dose; 0 h (within 30 min before dosing) on Days 2, 3, 4, 5, and 6; identical to those of the SAD group on Day 7. Metabolic transformation study: Ten participants from the food-effect group (Period 1, fasting condition) were included. All urine samples were collected within 2 h before dosing and from 0 to 72 h post-dose in Period 1. Fecal samples were also collected from 0 to 72 h post-dose.

This study evaluated several pharmacokinetic (PK) parameters following the initial dose, including the maximum observed plasma concentration (C_max_), the terminal elimination half-life in plasma (t_½_), time to reach peak concentration (T_max_), area under the concentration-time curve from time zero to the last measurable time point (AUC_0–t_), and the extrapolated area under the curve from time zero to infinity (AUC0– ∞ ). After multiple administrations, an additional analysis of the accumulation at steady state was performed. The plasma concentration-time profile of XT1061 was analyzed using non-compartmental methods with WinNonlin Professional software (Version 6.4, Pharsight Corporation, NC, USA).

### 2.4 XT1061 concentration in biological samples

The concentrations of XT1061 in plasma, urine, and feces were quantified by Suzhou Guochen Biotechnology Co., Ltd (Suzhou, China) using liquid chromatography coupled with tandem mass spectrometry (LC-MS/MS). All plasma samples were analyzed within their established stability period and fulfilled predefined quality acceptance standards. Plasma drug concentration measurements were performed in accordance with validated standard operating procedures. The calibration curve for XT1061 was linear over the range of 5.00 to 5,000 ng/mL.

### 2.5 Statistical analysis

Descriptive statistical methods were used. In the food-effect study, a mixed-effects analysis of variance (ANOVA) model was applied, incorporating subject as a random effect, to evaluate differences in C_max_ and AUC. The results were presented as geometric mean ratios and their 90% confidence intervals. To assess dose proportionality across the dose range of 12.5 mg–600 mg, SAS software (Version 9.4, SAS Institute Inc., Cary, NC, USA) was utilized, where C_max_ and AUC were analyzed using a linear mixed-effects model. Statistical results were presented as mean ± standard deviation or as frequency (percentage), as appropriate. A log-transformed independent-samples t-test was used to compare C_max_ and AUC values between male and female subjects in each group.

## 3 Results

### 3.1 Demographics

A total of 299 subjects were screened, and 90 subjects were enrolled. One subject withdrew before dosing of his own accord, while the remaining 89 subjects completed the trial and were included in the safety analysis set. The age of participants across all groups ranged from 35.8 to 47.8 years. Body mass index (BMI) values ranged from 22.6 to 24.8 kg/m^2^, with a comparable distribution of males and females (approximately 1:1). The majority of subjects were Han Chinese, with six being Hui. Demographic characteristics were well balanced across treatment groups (Table 1).

**TABLE 1:** Demographic characteristics of participants.

Baseline parameters	Single-ascending-dose study								Multiple-dose study	​
	12.5 mg (N = 8)	25 mg (N = 8)	37.5 mg (N = 8)	62.5 mg-A (N = 8)	62.5 mg-B (N = 8)	125 mg (N = 8)	250 mg (N = 8)	600 mg (N = 8)	250 mg (N = 8)	Placebo (N = 17)
Age, years	43.0 (9.7)	47.8 (9.0)	41.9 (10.4)	41.9 (12.3)	41.6 (7.9)	41.0 (5.9)	39.3 (7.8)	39.5 (5.5)	35.8 (7.5)	40.1 (9.0)
Gender: Male, n (%)	5 (62.50)	4 (50.00)	4 (50.00)	4 (50.00)	4 (50.00)	4 (50.00)	4 (50.00)	4 (50.00)	4 (50.00)	8 (47.06)
Ethnicity: Han, n (%)	8 (100.00)	8 (100.00)	7 (87.50)	7 (87.50)	8 (100.00)	8 (100.00)	7 (87.50)	8 (100.00)	8 (100.00)	14 (82.35)
Weight, kg	62.99 (7.05)	60.29 (5.02)	61.61 (9.35)	65.79 (8.17)	64.09 (9.60)	61.65 (8.78)	61.41 (5.49)	61.06 (8.85)	68.55 (11.90)	64.76 (9.95)
BMI, kg/m^2^	23.0 (1.5)	22.6 (2.3)	23.6 (1.8)	24.8 (2.1)	23.5 (2.7)	23.6 (3.0)	22.9 (1.6)	22.6 (2.6)	24.4 (2.8)	23.9 (2.7)

Data are presented as mean (standard deviation, SD), unless stated otherwise.

Subjects in the 62.5 mg-A, group received the study drug under the fasted condition first, followed by dosing in the fed state after a washout period. For the 62.5 mg-B, group, subjects were dosed in the fed state initially and then in the fasted state after washout. Each subject completed two dosing sessions.

### 3.2 PK profiles of XT1061

**FIGURE 2:**
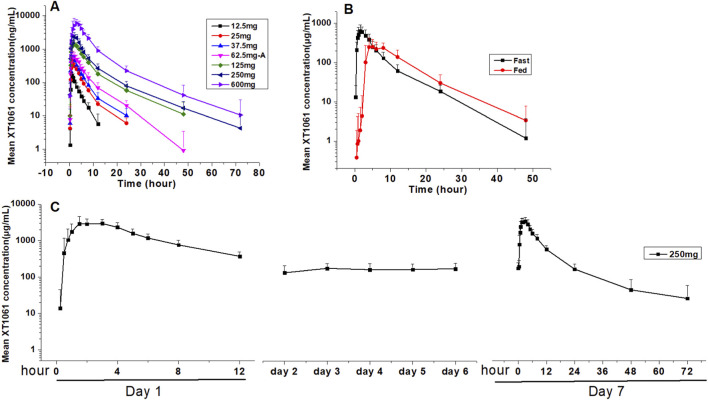
Mean plasma XT1061 concentration–time profiles in each treatment group for the single-ascending-dose study, **(A)** the food-effect study, **(B)** and the multiple-dose study. **(C)** Data are presented as mean ± standard deviation (SD).

The median T_max_ of XT1061 following single oral doses of 12.5–600 mg in healthy subjects under fasting conditions was 1.000–2.500 h; the mean plasma exposure (C_max_ and AUC) increased with dose; the mean t_1/2_ was 2.864–11.478 h. Pharmacokinetic parameters and the PK concentration–time profiles for each dose group are presented in Table 2 and Figure 2. The dose proportionality of XT1061 was assessed for C_max_, AUC_0-_ ∞ , and AUC_0-t_; the estimated slope (90% confidence interval (CI)) from the power model analysis was 0.95 (0.90–1.00), 1.09 (1.04–1.14), and 1.11 (1.05–1.16), respectively.

**TABLE 2:** Pharmacokinetic parameters of XT1061 after single-ascending-dose administration in each treatment group under the fasted state.

PK parameters	12.5 mg (N = 8)	25 mg (N = 8)	37.5 mg (N = 8)	62.5 mg-A (N = 8)	125 mg (N = 8)	250 mg (N = 8)	600 mg (N = 8)
T_max_ (h)	1.000 (0.75,1.50)	1.250 (0.75,3.00)	1.001 (0.75,2.00)	1.500 (0.75,2.00)	1.750 (1.00,3.00)	2.000 (1.00,3.00)	2.500 (1.50,4.00)
C_max_ (ng/mL)	162.375 (42.000)	408.250 (93.686)	610.750 (258.030)	701.500 (183.909)	1,655.000 (361.307)	2,790.000 (526.009)	7,436.250 (1780.706)
t_1/2_ (h)	2.864 (0.627)	4.519 (1.127)	4.459 (0.439)	6.149 (1.314)	8.092 (1.783)	11.478 (5.574)	8.957 (4.412)
AUC_0-_ ∞ (h*ng/mL)	561.384 (185.641)	1769.340 (227.680)	2,521.504 (965.316)	3,905.092 (1,248.693)	10191.504 (2,615.868)	15202.478 (3,048.591)	46206.529 (9,469.961)
AUC_0-t_ (h*ng/mL)	519.751 (174.488)	1720.367 (224.892)	2,454.734 (920.334)	3,761.355 (1,242.355)	9,944.457 (2,476.185)	14952.570 (3,036.198)	45800.157 (9,364.930)

Data are presented as mean (SD), or median (minimum, maximum).

62.5 mg–A, denotes the results obtained under the fasted state during Period 1 of the food effect study.

A high-fat meal can prolong the absorption of XT1061 (with the median T_max_ prolonged by approximately 4 h), reduce the average C_max_ (the mean postprandial C_max_ is 48.5% of that in the fasting state), but has little effect on AUC and t_1/2_. The geometric mean ratios of C_max_ and its 90% CI under fasting versus fed conditions were 2.11 (1.66, 2.69), and those of AUC_0-t_ and AUC_0-_ ∞ were 1.14 (0.99, 1.33) and 1.11 (0.95, 1.30), respectively. Thus, a high-fat meal has a significant impact on C_max_ but little effect on AUC (Supplementary Table 1; Figure 2).

After a single fasting oral administration of 62.5 mg XT1061 to 8 healthy subjects, the average cumulative excretion amounts of XT1061 in urine and feces within 72 h were 1.48 mg and 5.55 mg, respectively, with average cumulative excretion rates of 2.369% and 8.882%, respectively.

The average exposure of XT1061 (measured by AUC) increased after multiple administrations, but the accumulation was slight (with an average accumulation index of 1.333). The concentrations before administration on Day 2 to Day 7 indicated that a steady state could be achieved with once-daily administration for 2 consecutive days (Table 3; Figure 2).

**TABLE 3:** Pharmacokinetic parameters of XT1061 after multiple-dose administration of the 250 mg regimen (n = 8).

PK parameters	Day 1	Day 7
T_max_ (h)	1.501 (0.75, 3.01)	2.000 (1.50, 4.00)
C_max_ (ng/mL)	3,928.750 (845.973)	3,983.750 (800.445)
AUC_0-24h_ (h*ng/mL)	19251.027 (4,398.235)	25187.864 (4,949.466)
AUC_0-t_ (h*ng/mL)	19229.999 (4,389.186)	28445.002 (6,297.443)
AUC_0-_ ∞ (h*ng/mL)	20529.382 (5,184.368)	29442.055 (7,055.249)
t_1/2_ (h)	6.328 (1.544)	14.239 (10.892)
RAC (AUC_0-24h_)	​	1.333 (0.260)
RAC (C_max_)	​	1.043 (0.268)
C_min_ (ng/mL)	​	170.188 (69.658)

Data are presented as mean (SD), or median (minimum, maximum).

RAC, accumulation index.

In the SAD group, a statistically significant gender difference in AUC was observed in the 600 mg group (P < 0.05). For this group, the mean AUC_0-_ ∞ was 52,876 h*ng/mL in females—approximately 1.3-fold higher than that in males (39,536 h*ng/mL). The mean AUC_0-t_ was 52,308 h*ng/mL in females—also about 1.3-fold higher than that in males (39,291 h*ng/mL). No significant gender difference was found for C_max_. No gender-related differences in C_max_ or AUC were detected in the other dose groups. In the multiple-dose group, gender comparisons of C_max_ and AUC were conducted on Day 1 and Day 7 post-dose. Results indicated no significant gender differences in exposure parameters.

### 3.3 Safety and tolerability

Among the 89 subjects included in the safety analysis set, a total of 21 (23.60%, 21/89) experienced adverse events, of which 20 (22.47%, 20/89) had adverse events related to XT1061. Among the adverse events, the majority were CTCAE grade I, with 2 cases of grade II (both related to XT1061); no grade III or higher adverse events or adverse events leading to dropout occurred. Good tolerance was observed after single-dose administration of XT1061 at doses ranging from 12.5 mg to 600 mg or multiple-dose administration of 250 mg for 7 consecutive days. There was no significant difference in safety between the XT1061 dose groups and the placebo group, and no dose-related increase in the incidence of adverse events was observed (Table 4).

**TABLE 4:** Adverse events related to XT1061.

Drug-related adverse events	Single-ascending-dose study								Food effect study		Multiple-dose study	
	12.5 mg (N = 8)	25 mg (N = 8)	37.5 mg (N = 8)	62.5 mg-A (N = 8)	125 mg (N = 8)	250 mg (N = 8)	600 mg (N = 8)	Placobo (N = 14)	Fast	Fed	250 mg	Placebo
									(N = 16)	(N = 16)	(N = 8)	(N = 3)
Drug-related TEAEs	0	0	2 (25)	1 (12.5)	1 (12.5)	2 (25)	2 (25)	3 (21.43)	2 (12.5)	2 (12.5)	7 (87.5)	1 (33.33)
Hypophosphatemia	0	0	1 (12.5)	0	0	1 (12.5)	1 (12.5)	0	0	0	0	0
Chest discomfort	0	0	0	0	0	0	0	0	0	0	6 (75)	1 (33.33)
Elevated alanine aminotransferase	0	0	0	0	0	0	1 (12.5)	0	0	0	0	0
Elevated aspartate aminotransferase	0	0	0	0	0	0	1 (12.5)	1 (7.14)	0	0	0	0
Elevated amylase	0	0	1 (12.5)	0	0	0	0	0	0	0	0	0
Elevated blood uric acid	0	0	0	1 (12.5)	0	0	0	0	1 (6.25)	0	3 (37.5)	0
Elevated triglycerides	0	0	0	0	1 (12.5)	0	0	1 (7.14)	0	1 (6.25)	0	0
Elevated bilirubin	0	0	1 (12.5)	0	0	1 (12.5)	1 (12.5)	0	1 (6.25)	1 (6.25)	0	1 (33.33)
First-degree atrioventricular block	0	0	0	0	0	0	0	1 (7.14)	0	0	0	0

The data are presented as n (%). TEAE, treatment-emergent adverse event.

62.5 mg–A, denotes the results obtained under the fasted state during Period 1 of the food effect study.

Subjects in the 62.5 mg-A, group received the study drug under the fasted condition first, followed by dosing in the fed state after a washout period. For the 62.5 mg-B, group, subjects were dosed in the fed state initially and then in the fasted state after washout. Each subject completed two dosing sessions. Therefore, the food effect study comprised 16 subjects per subgroup under fasted and fed conditions.

In the SAD study, a total of 70 subjects were enrolled; 8 (14.29%, 8/56) in the treatment group experienced adverse events, all of which were judged to be drug-related. In the placebo group, 4 (28.57%, 4/14) experienced adverse events. The main adverse events in the SAD study were elevated bilirubin (1 case each in the 37.5 mg, 250 mg, and 600 mg groups, with a single-group incidence rate of 12.50%) and hypophosphatemia (1 case each in the 37.5 mg, 250 mg, and 600 mg groups, with a single-group incidence rate of 12.50%). The adverse events were mostly sporadic.

In the food effect study, a total of 16 subjects were enrolled; 2 (12.50%) in each of the fasting and fed groups experienced adverse events. Adverse events after administration in the fasting group included elevated uric acid (1 case) and elevated bilirubin (1 case); those in the fed group included elevated triglycerides (1 case) and elevated bilirubin (1 case).

In the multiple-dose study, a total of 11 subjects were enrolled; 7 (63.64%) in the treatment group experienced adverse events, all of which were drug-related. In the placebo group, 1 (33.33%) experienced an adverse event. The adverse events in the multiple-dose study included chest discomfort (75% in the treatment group vs. 33.33% in the placebo group), elevated uric acid (37.50% in the treatment group vs. 0% in the placebo group), and elevated bilirubin (0% in the treatment group vs. 33.33% in the placebo group).

Among all adverse events in this study, except for 2 cases of elevated triglycerides (grade II; 1 case each in the fed and placebo groups), all others were grade I. No intervention measures were taken, and all outcomes were “completely recovered without sequelae”.

## 4 Discussion

XT1061 single-dose administration at doses ranging from 12.5 mg to 600 mg or multiple-dose administration at 250 mg for 7 consecutive days was well tolerated, with all adverse events being mild. There was no significant difference in safety between the XT1061 dose groups and the placebo group, and no dose-related increase in the incidence of adverse events was observed. No significant changes were observed in important safety indicators—including ALT, AST, bilirubin, creatinine, uric acid, PR interval, heart rate, blood pressure, and QTc—before and after administration. The safety profile was similar to that of currently reported core protein allosteric modulators; that is, no dose dependency was observed in the incidence and intensity of adverse events (Zoulim et al., 2020; Jia et al., 2023; Jiang et al., 2023).

In the multiple-dose study, chest discomfort occurred in 75% of the treatment group and 33.33% of the placebo group, resulting in a higher incidence of adverse events in the treatment group. However, the severity of these adverse events was all grade I, and no clinically significant abnormalities were found in the electrocardiogram, physical examination, and laboratory tests of these subjects. The symptoms did not worsen after continued medication, and most were intermittent or transient, all of which recovered completely on their own. However, longer-term administration of XT1061 is still needed, and adverse events in this regard should be closely monitored.

In the safety analysis set, 21 of 89 subjects (23.6%) experienced adverse events, and 20 of these 21 subjects (95.2%) had adverse events considered related to XT1061. Although most of these events were mild in severity, the high proportion of treatment-related adverse events warrants attention. This finding suggests that while XT1061 was generally well tolerated in this study, careful safety monitoring remains necessary in subsequent Phase II and III trials, particularly for frequently occurring adverse events and any events of greater severity.

In this study, the pharmacokinetic (PK) behavior of XT1061 was evaluated in healthy subjects and was best described by a two-compartment model featuring first-order absorption (Zhang et al., 2021). The C_max_ and AUC of XT1061 exhibited dose-proportional increases across the tested range. XT1061 displays rapid initial elimination followed by a slower terminal phase, particularly evident at the 600 mg dose level. Extending the PK sampling duration—from 0–24 h on day 1 to 0–72 h on day 7—resulted in a longer apparent t1/2 (Zhang et al., 2021), which explains the observed increase in half-life following multiple dosing, rising from 6.328 to 14.239 h.

Additionally, the study demonstrated that food intake did not affect the overall systemic exposure (AUC) of XT1061, although it reduced the peak concentration. This indicates that food is unlikely to compromise the therapeutic efficacy of XT1061, suggesting no need for strict dietary restrictions during treatment (Karalis et al., 2008; Zhang et al., 2018).

The plasma exposure level of XT1061 in the 37.5–600 mg dose groups at 12 h and 24 h after dosing was 33–907 ng/mL and 10–224 ng/mL, respectively. The EC50 value of XT1061 in the HepG2.2.15 cell line was 1.04 nmol/L, which corresponds to 7.6 ng/mL after correction for plasma protein binding (93.2%). Therefore, a 37.5 mg dose in humans can achieve antiviral activity against HBV DNA comparable to that observed in the *in vitro* study (Zhang et al., 2021). In the mouse efficacy model, the effective dose of XT1061 monotherapy was 50 mg/kg, which translates to approximately 267 mg in humans (data not published). In this study, single-ascending doses of 12.5–600 mg and multiple-dose administration of 250 mg were safe and well tolerated. Thus, if 250 mg is used in future clinical studies in patients with CHB, it is theoretically safe and effective (Yao et al., 2025). Accordingly, a dose of approximately 250 mg of XT1061 is recommended for the Phase Ib clinical study in CHB patients. This study is a short-term evaluation of safety, tolerability, and pharmacokinetics conducted in healthy subjects, and has not yet yielded results on long-term antiviral efficacy or related toxicities. Future antiviral studies in chronic HBV-infected patients are needed to confirm the drug’s efficacy and long-term safety.

## 5 Conclusion

This study demonstrated that XT1061 has a safe and well-tolerated profile, along with favorable pharmacokinetic properties, providing valuable information to support future clinical trials evaluating its safety and efficacy in patients with CHB.

## Data Availability

The raw data supporting the conclusions of this article will be made available by the authors, without undue reservation.
