# Supplementary material for: First-in-human study on the pharmacokinetics, safety, and tolerability of single escalating doses and multiple doses of XT1061, a novel core protein allosteric modulator, in healthy Chinese subjects
Source: Front Pharmacol. 2026 Jun 30;17:1860210. doi: 10.3389/fphar.2026.1860210 (PMC13365723; doi:10.3389/fphar.2026.1860210)
Supplement: Supplementary file 1 [file Table1.docx]

**Supplementary Table 1. Analysis of the pharmacokinetic effects of a high-fat meal.**

|  | Fast (N = 16) | Fed (N = 16) | Ratio (Fast versus Fed, %) |
| --- | --- | --- | --- |
| T_max_ (h) | 1.500 (0.75, 3.00) | 5.500 (3.00, 12.00) | |
| C_max_ (ng/mL) | 730.938 ± 229.919 | 354.500 ± 137.891 | 2.11(1.66,2.69) |
| AUC_0-t_ (h*ng/mL) | 3611.108 ± 1093.443 | 3208.333 ± 1052.745 | 1.14(0.99,1.33) |
| AUC_0-∞_ (h*ng/mL) | 3737.247 ± 1110.861 | 3352.709 ± 1140.968 | 1.11(0.95,1.30) |
| t_1/2_ (h) | 6.546 ± 3.144 | 6.144 ± 1.807 | |

Data are expressed as mean ± SD or median (minimum, maximum).
